# Proficiency and Difficulty Scoring Tools for Finger Replantation

**DOI:** 10.1001/jamanetworkopen.2025.40453

**Published:** 2025-10-30

**Authors:** Kevin C. Chung, Adee Heiman, Sunitha Malay, and the Proficiency and Difficulty Scoring Tools for Finger Replantation (PRAISE) Study Group, Emily R. Geis, Lu Wang, Alfred P. Yoon, Trista M. Benitez, Zihao Han, Aviram M. Giladi, Isaac C. Fleming, Daniel J. Gray, Nicole A. Zelenski, Michael B. Gottschalk, Eric R. Wagner, Karishma R. Desai, Alessio M. Griffin, S. Raja Sabapathy, R. Raja Shanmugakrishnan, Shruthi Chandrasekar, Joshua M. Adkinson, Robin E. Gardiner, Soumen Das De, Sandeep J. Sebastin, Hima Premnadh, Jocelyn Teo, Sonu A. Jain, Kara Colvell, Phillip R. Ross, Adam C. Valenti

**Affiliations:** 1 Section of Plastic Surgery, University of Michigan Medical School, Ann Arbor; 2 Section of Plastic Surgery, Michigan Medicine, Ann Arbor; 3 Michigan Medicine, Ann Arbor; 4 Now at UC Davis Health, University of California, Davis; 5 Medstar Union Memorial Hospital, Baltimore, Maryland; 6 Emory University, Atlanta, Georgia; 7 Ganga Hospital, Coimbatore, Tamilnadu, India; 8 Indiana University, Indianapolis; 9 National University Hospital, Singapore, Singapore; 10 The Ohio State University, Columbus, OH; 11 Now at University of Cincinnati, Cincinnati, Ohio; 12 University of Cincinnati, Cincinnati, Ohio

## Abstract

**Importance:**

Replantation and revascularization are among the most technically challenging surgical procedures and have varying success rates. Methods to assess surgeon skill for this complex undertaking are lacking.

**Objective:**

To examine whether a surgeon proficiency score is associated with clinical outcomes across multiple institutions.

**Design, Setting, and Participants:**

This multicenter retrospective cohort study analyzed all digit replantation and revascularization after traumatic amputations for adult patients from January 1, 2000, to August 31, 2022, at 8 medical institutions in the United States, India, and Singapore. Data were collected and analyzed from February to October 2024.

**Exposure:**

Surgeon proficiency (range, 1.0 to 7.5; higher score indicates greater proficiency) and procedure difficulty (range, −1.0 to 7.5; higher score indicates greater difficulty) scores. Each surgeon’s procedures were sorted chronologically, then divided in half. Proficiency scores were calculated for each surgeon based on the difficulty and outcomes of their early procedures and validated using outcomes from their later procedures. Difficulty scores were calculated based on patient and injury characteristics.

**Main Outcomes and Measures:**

The outcome of interest was procedure success, defined as digit survival at 1 month after the operation.

**Results:**

Of 653 digits that were replanted or revascularized by 65 surgeons, 458 (70.1%) were successful. Mean (SD) patient age was 41.0 (15.7) years, and 571 patients (87%) were male. Difficulty scores ranged from 1.0 to 7.5, with a mean of 2.7, and were significantly higher in failed procedures than in successful procedures (mean [SD], 3.2 [1.4] vs 2.5 [1.2]; *P* < .001). Proficiency scores ranged from −0.37 to 4.14, with a mean of 1.40. For each 1-point increase in proficiency score, a surgeon’s future success rate increased by 7.5% (95% CI, 5.5%-8.1%; *P* < .001). After adjusting for surgeon experience, difficulty score, and additional patient- and injury-level characteristics using mixed-effects logistic regression, greater surgeon proficiency score and lower difficulty score were associated with greater likelihood of individual procedure success (surgeon proficiency: odds ratio [OR], 1.46; 95% CI, 1.02 to 2.10; *P* = .04; difficulty: OR, 0.70; 95% CI, 0.57 to 0.86; *P* = .001).

**Conclusions and Relevance:**

This multi-institutional cohort study of patients who underwent digit replantation or revascularization found that surgeon proficiency score was associated with 1-month success. These findings suggest that national trauma referral networks should incorporate difficulty and proficiency scoring tools when coordinating patient transfers after traumatic digit amputation. Centers with high replant volume or those who are geographically well-positioned to become replant centers should aim to recruit and improve reimbursement for surgeons with high proficiency scores. Meanwhile, surgeons with low proficiency scores should seek out additional training to improve their skills.

## Key Points

**Question:**

Is surgeon proficiency score associated with 1-month success after replantation of an amputated digit?

**Findings:**

This cohort study including 653 digit replantation or revascularization procedures found an association between surgeon proficiency score, calculated by a previously developed scoring model, and digit survival outcomes at 1 month.

**Meaning:**

These findings suggest that whenever feasible, patients with difficult replantation procedures should be triaged to greater-proficiency surgeons to maximize survival and optimize resource use.

## Teaser

This cohort study examines the association of a surgeon proficiency score with digit replantation and revascularization outcomes across 8 tertiary care institutions in the US, India, and Singapore.

## Introduction

The volume-outcome association has long been acknowledged within the surgical community, since a 1979 study reported lower mortality rates in surgical procedures performed at high-volume hospitals.^1^ Numerous publications have detailed how high-volume hospitals have improved outcomes and potentially decreased costs after complex procedures, including pancreaticodueodenectomy,^2,3^ radical prostatectomy,^4^ bariatric surgery,^5^ and esophagectomy.^6^ This has resulted in widespread calls for regionalization of these procedures to ensure that patients are treated by the most experienced surgeons. However, volume is only a single contributor to surgeon skill and may not explain the full variation in performance. Birkmeyer et al^7^ were the first to grade bariatric surgeon technique using video assessment. Multiple studies have reported that surgeons who score well on video-based assessments or simulations of technically demanding procedures demonstrate improved complication profiles.^7,8,9,10,11,12,13,14,15^

One major challenge is assessing surgeon skill in emergent surgical procedures that are not amenable to video assessment. Digit replantation and revascularization after prevalent traumatic amputation are technically challenging procedures with varying success rates (48%-97%).^16^ These injuries result in functional impairment, loss of work or hobbies, and decreased quality of life. A successful result can potentially improve the function and aesthetics of the hand.^17,18^ However, surgeons and medical centers with a track record of failure lead to substantial financial waste.^19^

For this reason, Yoon et al^20^ developed a Surgeon Proficiency Score (SPS) grading model to measure a surgeon’s replant capabilities based on the difficulty and success of their prior procedures. Using data from a single institution, they found that SPS was more strongly associated with 1-month success and complications than experience or prior volume.^20^ This objective and easy-to-calculate metric is the first surgeon grading system within trauma surgery to be validated by clinical data, to our knowledge. It can be used to track surgeon progress, influence referral networks for difficult procedures, recruit well-qualified surgeons to high-volume replant centers, and identify surgeons who would benefit from additional training or mentorship. The purpose of this study was to validate this scoring system across multiple international institutions. The concepts developed from this study can facilitate surgeon proficiency assessment for complex surgeries as part of the trauma center accreditation process.

## Methods

The Proficiency and Difficulty Scoring Tools for Finger Replantation (PRAISE) study was approved by the University of Michigan institutional review board with a waiver of informed consent because due to the retrospective nature of the study. The PRAISE study is a multi-institutional retrospective cohort study funded by the American Foundation for Surgery of the Hand. This study followed the Strengthening the Reporting of Observational Studies in Epidemiology (STROBE) reporting guideline for retrospective cohort studies. Health record review was performed on all patients who underwent attempted digit replantation or revascularization after traumatic amputation between January 1, 2000, and August 31, 2022. Data were collected in 2024 from 8 tertiary care institutions with replantation capabilities in the US, India, and Singapore. Participating centers were selected based on amputation case volume and prior collaboration with the principal investigator at the coordinating center. All but 1 of the hospitals were located in cities with at least 2 million people. There were no uniform indications for determining whether or not replantation or revascularization was attempted; this was based on clinical assessment and discussion with the patient. We excluded patients younger than 18 years, digits converted to revision amputation intraoperatively, and digits that were revascularized without preoperative signs of ischemia. If a patient had amputation of multiple digits, we only included digits amenable to replantation or revascularization. *Revascularization* was defined as repair of a partially amputated digit with vascular compromise but an intact skin bridge, whereas *replantation* refers to a completely amputated digit.

### Data Collection

Each participating center obtained their own institutional review board approval. The coordinating center conducted a virtual training session with research teams at the participating centers. A data collection sheet was provided that included all variables to ensure consistency. Centers were instructed to include all replantation and revascularization procedures performed at their facility. Data were collected from the electronic medical record. Patient-level variables included age, sex, smoking status, and number of Elixhauser comorbidities. Injury-level variables included procedure type (replantation vs revascularization), affected digit(s), mechanism of injury (sharp, crush, or avulsion), ischemia time (<12 or ≥12 hours), and zone of injury. We also collected data on each surgeon’s years of experience and total number of replantation or revascularization procedures they performed. Because of inconsistencies in reporting total number of procedures between institutions, only procedures reported in this study were included. We defined 1 procedure as a single digit (meaning that if 2 digits were replanted during the same surgery, they were classified as 2 different procedures). Our primary outcome was procedure success, defined as digit survival at 1 month, by which point a failed digit will have mummified. Our secondary outcome was total complications. Complications included stiffness, nonunion, severe infection, and the need for revision surgeries. Surgeons were excluded from the study if they only performed a single procedure or if they were not attending physicians.

### Statistical Analysis

#### Bivariate Analysis of Characteristics

Patient- and surgeon-level characteristics of successful and failed procedures were compared using unadjusted analysis. We used *t* test for continuous variables and χ^2^ for categorical variables).

#### Procedure Difficulty Score Calculation

In a 2021 study, Yoon et al^20^ calculated pooled relative risks for factors associated with failure from a meta-analysis of 31 studies and identified 5 risk factors for failure: procedure type (replantation vs revascularization), smoking status (smoker vs nonsmoker), number of digits (multiple vs single), mechanism of injury (crush vs sharp, avulsion vs sharp), and zone of injury (Tamai zones 1/2 and T1/T2 vs other).^20^ We conducted an updated literature search and identified 25 additional studies reporting outcomes of revascularization and replantation and added data from these studies into the pooled relative risks.^21,22,23,24,25,26,27,28,29,30,31,32,33,34,35,36,37,38,39,40,41,42,43,44,45^ We also added data from procedures that were excluded from our study. New pooled relative risks are shown in Table 1. Difficulty scores for each digit were calculated by multiplying the relative risk for factors corresponding to patient and injury characteristics. For example, a smoker who experiences a crush injury resulting in complete amputation of the middle finger at zone 3 (requiring replantation) and partial amputation of the index finger at zone 2 (requiring revascularization) would receive the following scores:

Index finger: 1 (revascularization) × 1.22 (smoker) × 1.49 (multidigit) × 1.16 (crush) × 1.12 (zone 2) = 2.36;

Middle finger: 2.03 (replantation) × 1.22 (smoker) × 1.49 (multidigit) × 1.16 (crush) × 1 (zone 3) = 4.28.

We used *t* test to compare difficulty scores between successful and failed procedures.

**Table 1.** Relative Risks

Covariate	Pooled relative risk
Procedure (replantation vs revascularization)	2.03
Smoking status (smoker vs nonsmoker)	1.22
Number of digits (1 vs multidigit)	1.49
Mechanism of injury	
Crush vs sharp	1.16
Avulsion vs sharp	1.82
Zone of injury (Tamai zones 1/2 vs others)	1.12

#### SPS Calculation

Each surgeon’s total case load was split in half chronologically, with the first half consisting of the surgeon’s earlier procedures and the second half consisting of their later procedures. SPS was then calculated based on each half:

(Σ [difficulty score of successful procedures] – Σ [1 / difficulty score of failed procedures]) / total number of procedures.

If a surgeon performed an odd number of procedures, the median procedure was used as a test procedure.

The correlations among each surgeon’s first-half SPS, years of experience, and overall case volume were tested using Pearson correlation coefficient. This was also used to check for collinearity between surgeon experience and volume.

#### SPS Validation

Pearson correlation coefficient was used to assess the correlation between each surgeon’s first-half SPS and success rates of the second-half procedures. Mixed-effect logistic regression was used to evaluate the association between early SPS and later success, adjusting for the surgeon’s years of experience at the time of each procedure, difficulty score, and the following patient- and injury-level factors: patient age, sex, Elixhauser comorbidities, thumb vs nonthumb amputation, and ischemia time. A mixed-effect Poisson regression model was used to evaluate the association between SPS and total complications per procedure, with similar adjustments.

Statistical analyses were performed using R software version 4.2.1 (R Project for Statistical Computing) and Stata version 18 (StataCorp). The statistical significance level was defined as 2-sided α = .05. Data were collected and analyzed from February to October 2024.

#### Missing Data

If success vs failure of replantation was not reported due to loss of follow-up (which occurred with 12 digits), it was assumed that the procedure was successful because it is inconceivable that the patient will live with a mummified digit. Symptoms of digital necrosis will be evident enough that the vast majority of patients will seek medical attention for this. (One center with approximately 30% loss to follow-up reported that patients were not expected to follow up unless they experienced complications. This is a safety-net hospital with a high volume of uninsured patients who often do not follow up because of financial or other socioeconomic barriers.) We excluded patients who were lost to follow-up (7 patients in the second half of the cohort) from the model examining complications. If a variable required to calculate difficulty score was missing, multiple imputation was used to fill in the missing variable.

## Results

### Patient and Procedure Characteristics

Data on 500 adult patients who underwent replantation or revascularization of 710 digits by 90 surgeons were collected. After excluding procedures performed by residents or by surgeons who had only performed a single procedure, 653 digits and 65 surgeons met inclusion criteria. A total of 571 procedures (87%) were performed in male patients. Mean (SD) age was 41.0 (15.7) years. There were 332 replantations and 318 revascularizations performed. The overall 1-month success rate was 458 procedures (70%).

Table 2 shows patient-, injury-, and surgeon-level variables for overall, successful, and failed digits. On unadjusted analysis, the factors significantly associated with improved success were revascularization vs replantation (264 successes [83%] vs 191 successes [58%]; *P* < .001), being a nonsmoker vs former smoker (221 successes [70%] vs 28 successes [56%]; *P* = .04), nonthumb amputation (378 successes [73%] vs 80 successful thumb procedures [58%]; *P* < .001), and prior case volume (mean [SD], 9.1 [9.3] procedures for surgeons who performed successful procedures vs 6.5 [7.6] procedures for surgeons who performed failed procedures; *P* < .001).

**Table 2.** Patient-, Injury-, and Surgeon-Level Variables for Total, Successful, and Failed Digits

Variable	Total, No.	No. (%)		*P* value^a^
		Successful	Failed	
Digits	653	458 (70)	195 (30)	
Age, mean (SD), y	41.0 (15.7)	41.2 (15.9)	40.4 (15.4)	.58
Sex				
Male	571	402 (70)	169 (30)	.70
Female	82	52 (63)	26 (37)	
Procedure				
Replantation	332	191 (58)	141 (42)	<.001
Revascularization	318	264 (83)	54 (17)	
Unknown	3	3 (100)	0	NA
Smoking status				
Never	314	221 (70)	93 (30)	NA
Former	50	28 (56)	22 (44)	.04^b^
Current	202	136 (67)	66 (33)	.46^b^
Unknown	87	73 (84)	14 (16)	NA
Multidigit				
Yes	375	254 (68)	121 (32)	.12
No	278	204 (73)	74 (27)	
Injury mechanism				
Sharp	327	227 (69)	100 (31)	NA
Crush	222	164 (74)	58 (26)	.26^c^
Avulsion	104	67 (64)	37 (36)	.34^c^
Zone of Injury				
Distal				
Overall	529	377 (71)	152 (29)	.09
Zone 1	41	NA	NA	
Zone 2	387	NA	NA	
T1	37	NA	NA	
T2	64	NA	NA	
Proximal				
Overall	117	74 (63)	43 (37)	
Zone 3	87	NA	NA	
T3	28	NA	NA	
T4	2	NA	NA	
Unknown	7	7 (100)	0	NA
Affected Digit				
Thumb	138	80 (58)	58 (42)	<.001
Other	515	378 (73)	137 (27)	
Ischemia time, h				
<12	513	354 (69)	159 (31)	.25
≥12	135	100 (74)	35 (26)	
Unknown	5	4 (80)	1 (20)	
Prior case volume, mean (SD), No.	8.3 (8.9)	9.1 (9.3)	6.5 (7.6)	<.001
Surgeon experience, mean (SD), y	10.7 (7.8)	11.0 (7.7)	10.2 (8.0)	.21

Abbreviation: NA, not applicable.

^a^ Categorical variables are compared by χ^2^ test. Continuous variables are compared by *t* test.

^b^ Compared with never smokers.

^c^ Compared with sharp injury.

### Hospital Characteristics

Replantation and revascularization volume at each hospital ranged from 3.5 to 22.8 procedures annually (mean, 7.7 procedures; median, 4.8 procedures). All 11 surgeons from 1 hospital were excluded because all procedures were performed by residents. The remaining 7 hospitals had 7 to 18 attending surgeons performing replantation (mean, 11.3 surgeons; median, 11 surgeons). These surgeons performed a total of 667 procedures, ranging from 1 to 48 procedures per surgeon (mean, 8.4 procedures; median, 5 procedures).

### Difficulty Score

The mean (SD) difficulty score was 2.7 (1.3) (range, 1.0-7.5). Mean (SD) difficulty score for successful procedures was 2.5 (1.2), compared with 3.2 (1.4) for failed procedures. Difficulty scores were significantly higher in failed procedures (*P* < .001).

### SPS Calculation

The mean (SD) SPS for the first half of each surgeon’s procedures was 1.40 (1.00) (range, −0.37 to 4.14). This was not significantly different from the second half (mean [SD], 1.49 [1.00]; range, −0.44 to 4.15) by *t* test (*P* = .18) (eTable 1 in Supplement 1). Pearson correlation coefficient showed a weak but significant correlation between each surgeon’s first-half and second-half SPS (*r* = 0.27; *P* = .03).

Pearson correlation coefficient showed significant correlations between SPS and prior case volume (*r* = 0.32; *P* = .008), but did not indicate significance between SPS and years of experience (*r* = −0.17; *P* = .19). Years of experience and prior volume were very strongly correlated with each other (*r* = 0.37; *P* < .001). Because of inconsistencies in the reporting of case volume among the institutions and its high degree of collinearity with years of experience, prior case volume was removed from further analyses.

### SPS Validation

Using Pearson correlation coefficient, we found a significant correlation between first-half SPS and second-half success rates (*r* = 0.70; *P* < .001). For each 1-point increase in SPS, a surgeon’s future success rate increased by 7.5% (95% CI, 5.5%-8.1%; *P* < .001) (Figure). This association remained statistically significant after adjusting for surgeon experience, difficulty score, and additional patient- and injury-level characteristics that were not included in the difficulty score calculation using mixed-effects logistic regression. In this model, each 1-point increase in SPS was associated with 46% increased odds of success (odds ratio [OR], 1.46; 95% CI, 1.02 to 2.10; *P* = .04), while a 1-point increase in difficulty score was associated with 30% reduced odds of success (OR, 0.70; 95% CI, 0.57 to 0.86; *P* = .001) (Table 3). By modeling the total complication counts with a mixed-effect Poisson model, we found that complications were not significantly associated with any of the previously mentioned risk factors (Table 4; eTable 2 in Supplement 1).

**Figure.**
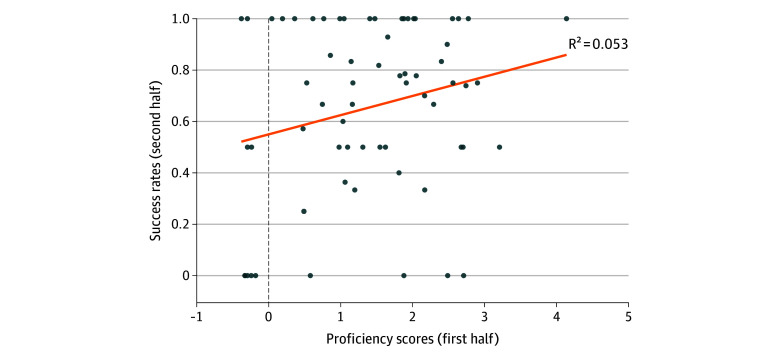
Surgeon Proficiency Scores vs 1-Month Success Rates

**Table 3.** Logistic Mixed-Effects Model: Factors Associated With Surgical Success With Difficulty Score

Factor	Fixed-effects estimates		
	Estimate (SE)^a^	*P* value	Odds ratio (95% CI)
Intercept	0.792 (0.677)	.24	2.21 (0.59 to 8.33)
Proficiency Score	0.378 (0.185)	.04	1.46 (1.02 to 2.10)
Difficulty Score	−0.36 (0.108)	.001	0.70 (0.57 to 0.86)
Age	0.019 (0.01)	.06	1.02 (1.00 to 1.04)
Sex	−0.581 (0.388)	.13	0.56 (0.26 to 1.2)
Elixhauser Score	−0.099 (0.156)	.53	0.91 (0.67 to 1.23)
Thumb	−0.718 (0.36)	.046	0.49 (0.24 to 0.99)
Ischemia time	0.395 (0.376)	.29	1.48 (0.71 to 3.1)
Years of experience	−0.001 (0.021)	.97	1 (0.96 to 1.04)

^a^ Estimates are log-odds.

**Table 4.** Mixed-Effects Poisson Model for Factors Associated With Complications

Factor	Fixed-effects estimate	
	Estimate (SE) [95% CI]^a^	*P* value
Intercept	−0.508 (0.308) [−1.111 to 0.095]	.10
Proficiency Score	0.052 (0.084) [−0.111 to 0.216]	.53
Difficulty Score	0.081 (0.047) [−0.011 to 0.173]	.08
Age	−0.002 (0.004) [−0.011 to 0.006]	.64
Sex	0.214 (0.169) [−0.117 to 0.546]	.21
Elixhauser Score	0.026 (0.07) [−0.111 to 0.162]	.71
Thumb	−0.076 (0.178) [−0.425 to 0.274]	.67
Ischemia time	0.01 (0.164) [−0.311 to 0.332]	.95
Years of experience	0.006 (0.009) [−0.012 to 0.024]	.51

^a^ Estimates are log-counts.

## Discussion

This cohort study used surgeon proficiency score as an indirect yet easy-to-calculate metric of surgeon skill based on the difficulty of a surgeon’s prior procedures and their track record of successes and failures. We aimed to validate findings from the single-institution study by Yoon et al,^20^ which found that proficiency scores were more strongly associated with digit survival than a surgeon’s experience or prior case volume. In this international study, we found that both lower difficulty score and greater SPS were associated with increased odds of success. We also found a significant association between SPS calculated from the first half and the second half of a surgeon’s procedures, suggesting that early operative proficiency correlates with later proficiency.

Several studies have reported an association between volume and replant success, prompting a trend toward increased regionalization of these services. A nationwide database study by Hustedt et al^46^ found greater replant success rates among high-volume surgeons at high-volume hospitals.^46^ A multistate database study by Brown et al,^47^ a study by Mahmoudi and Chung^48^ focusing on thumb replantation, and a geospatial analysis by O’Brien et al^49^ all reported greater success at high-volume hospitals. However, other studies have reported either mixed results or no association between success and case volume. A 2018 study by Cho et al^50^ found no association between hospital volume and survival. More recently, a 2021study by Hsu et al^51^ found lower success rates at high-volume hospitals in Taiwan but improved success associated with high-volume surgeons. This discrepancy might be explained by factors that are not measured in large database studies: procedure difficulty and technical skill.

Multiple studies have assessed surgeon skill through video assessments of laparoscopic and robotic procedures. Of these, 4 studies^7,11,12,14^ have included surgeon experience or case volume in their analysis. A study by Birkmeyer et al^7^ on gastric bypass found higher rates of certain complications after procedures performed by the lowest-scoring group. This group had lower annual procedure volume compared with groups with higher scores, but there was no difference in years of experience. None of the other studies reported case volume or surgeon experience as a significant contributor to assessment score or complication rate.^7^ Heard et al^11^ reported that greater surgeon score on radical prostatectomy was positively associated with recovery of erectile function, whereas surgeon caseload was not. Fecso et al^12^ found that lower scores on gastric cancer surgery were associated with major complications and noted multiple technical errors that led to intraoperative bleeding and tissue injury. There was no significant difference in surgeon experience between procedures that did or did not lead to major complications.^12^ Similarly, a study by Varban et al^14^ found that surgeons who received low scores on sleeve gastrectomy had higher rates of postoperative obstruction and hemorrhage, but there was no significant difference in years of experience between high- and low-scoring surgeons.^14^

In the case of digit replantation, direct measurement of surgeon skill through video assessment is rarely feasible because of the logistics of emergency surgery and the infrequent and unpredictable nature of these procedures. SPS serves as a substitute metric. Although it is based on relative risks specific to replantation failure, the ease of calculation means that similar metrics could be developed for other types of emergent procedures.

Another important takeaway from our study is how to incorporate surgeon proficiency data to improve structures and processes within the hand trauma system and in other specialized surgical fields. Future centralization of replantation services will ensure that more patients are treated at high-volume centers by experienced surgeons working within systems that are optimized for expedient, knowledgeable, and well-coordinated care.^52^ The American College of Surgeons’ most recent edition of *Resources for the Optimal Care of the Injured Patient* states that all level I and II trauma centers must have available replantation services at all times or a triage and transfer process with a center that does offer these services.^53^ Approximately half of all level I trauma centers and one-quarter of level II centers in the US offer replantation services.^29^ To demonstrate adherence to these recommendations, replant centers must maintain records of hand surgeon call schedules.^53^ However, there is no oversight or recommendations regarding the technical capabilities or quality of care provided by these surgeons. This is a significant shortfall, given the technical challenges of digital replantation and revascularization, where the small diameter of the vessels and the traumatized nature of the tissue mean that gentle handling and precise, controlled movements are critical. Replantation centers are responsible for ensuring the technical competence of their surgeons, so that the time and resources needed to transport patients are put to effective use. High-volume replant centers or hospitals in areas that are geographically well-positioned for replantation^54^ should aim to recruit and adequately reimburse high-proficiency surgeons. Meanwhile, surgeons whose scores remain consistently low after several procedures should reconsider performing replantation or seek out further training to improve their skills.

SPS and difficulty score can also be used to assist in patient triage. An uncomplicated injury, such as a partial amputation in a nonsmoker who severed both digital arteries while cutting a bagel, may well be within the capabilities of a surgeon with mid-range proficiency at a low-volume center. However, a musician with a mangled hand after a crush injury would benefit from transfer to a high-volume replantation center for evaluation by a high-proficiency surgeon.

### Limitations

This study has some limitations. We were primarily limited by the retrospective nature of the study and the lack of uniform indications for replantation; a procedure that might have been attempted by one surgeon may have been refused by another. This may explain why thumb replantation was associated with higher risk of failure: given the functional importance of this digit, surgeons may have been more likely to attempt a difficult thumb replant. Retrospective data and lack of randomization is a major limitation in the replantation literature, which also affects our ability to calculate relative risks and difficulty scores. We were also limited by missing data, including smoking and follow-up information. Additionally, the heterogeneity of each surgeon’s case count is another limitation, in that surgeons who performed only 2 procedures were compared against surgeons who performed up to 48 procedures. Future studies will focus on the question of how proficiency scores change over time and determining how many procedures a surgeon should perform before their score can be effectively measured.

## Conclusions

In this multi-institutional cohort study of replantation or revascularization surgical procedures, we found that SPS was associated with 1-month success. Our study has important policy implications. By identifying high-proficiency surgeons, we can improve referral networks within each region, increase recruitment of skilled surgeons, and ensure that patients with the most challenging injuries are sent to the highest levels of hand trauma care.

## Notes

Supplement 1. **eTable 1.** Case volumes, failures, success rates, average difficulty scores, and SPS from the first and second half of each surgeon’s cases **eTable 2.** Frequency of complications overall and in the second half of each surgeon’s cases

Supplement 2.
Data Sharing Statement
