# Supplementary material for: Proficiency and Difficulty Scoring Tools for Finger Replantation
Source: JAMA Netw Open. 2025 Oct 30;8(10):e2540453. doi: 10.1001/jamanetworkopen.2025.40453 (PMC12576492; doi:10.1001/jamanetworkopen.2025.40453)
Supplement: Supplement 1. — eTable 1. Case volumes, failures, success rates, average difficulty scores, and SPS from the first and second half of each surgeon’s cases eTable 2. Frequency of complications overall and in the second half of each surgeon’s cases [file jamanetwopen-e2540453-s001.pdf]

Supplemental Online Content

Chung KC, Heiman A, Malay S; the Proficiency and Difficulty Scoring Tools for Finger Replantation (PRAISE) Study Group. Proficiency and difficulty scoring tools for finger replantation. *JAMA Netw Open*. 2025;8(10):e2540453. doi:10.1001/jamanetworkopen.2025.40453

eTable 1. Case volumes, failures, success rates, average difficulty scores, and SPS from the first and second half of each surgeon's cases

eTable 2. Frequency of complications overall and in the second half of each surgeon's cases

This supplemental material has been provided by the authors to give readers additional information about their work.

© 2025 Chung KC et al. *JAMA Network Open*.

Supplemental Table 1. Case volumes, failures, success rates, average difficulty scores, and SPS from the first and second half of each surgeon's cases.

	First half of case series					Second half of case series				
**Surgeon**	**Total case volume (n = 344), No.**	**Failed (n = 103), No.**	**Success rate, %**	**Average procedure difficulty**	**Surgeon Proficiency Score**	**Total case volume (n = 319), No.**	**Failed (n = 88), No.**	**Success rate, %**	**Average procedure difficulty**	**Surgeon Proficiency Score**
1	4	3	25.0	4.2	0.5	4	3	25.0	4.8	0.7
2	3	1	66.7	2.3	1.3	2	1	50.0	4.9	3.5
3	8	5	37.5	3.4	1.1	8	4	50.0	3.0	1.2
4	4	1	75.0	3.2	2.6	3	0	100.0	3.1	3.1
5	4	0	100.0	2.6	2.6	4	1	75.0	4.2	2.9
6	6	2	66.7	2.7	1.5	5	0	100.0	2.4	2.4
7	1	0	100.0	1.9	1.9	1	0	100.0	1.0	1.0
8	2	1	50.0	2.8	1.0	1	0	100.0	2.3	2.3
9	2	1	50.0	3.4	1.5	2	1	50.0	3.1	1.8
10	1	1	100.0	3.4	-0.3	1	1	0.0	3.4	-0.3
11	9	3	66.7	2.5	1.2	9	3	66.7	3.5	2.4
12	1	1	0.0	3.4	-0.3	1	0	100.0	3.0	3.0
13	6	2	66.7	1.9	1.0	5	0	100.0	1.4	1.4
14	8	3	62.5	2.5	0.9	7	1	85.7	1.8	1.3
15	3	2	33.3	3.8	1.2	3	2	33.3	3.4	0.7
16	2	2	0.0	3.4	-0.3	2	1	50.0	3.2	1.3
17	3	2	33.3	2.8	0.7	3	1	66.7	3.4	2.2
18	4	0	100.0	2.9	2.9	4	1	75.0	3.3	2.8
19	11	1	90.9	1.6	1.4	11	0	100.0	2.1	2.1
20	5	1	80.0	1.7	1.2	4	1	75.0	1.8	1.0
21	4	3	25.0	1.5	-0.2	4	2	50.0	1.7	0.5
22	3	2	33.3	1.9	0.2	2	0	100.0	1.7	1.7
23	4	2	50.0	1.9	0.5	4	1	75.0	3.1	2.1
24	4	3	25.0	2.5	0.0	3	0	100.0	1.5	1.5

© 2025 Chung KC et al. *JAMA Network Open*.

25	1	1	0.0	2.6	-0.4	1	0	100.0	2.0	2.0
26	9	6	33.3	2.9	1.0	8	4	50.0	3.4	1.5
27	5	1	80.0	2.3	1.9	5	0	100.0	1.9	1.9
28	1	1	0.0	5.5	-0.2	1	1	0.0	2.3	-0.4
29	20	2	90.0	2.0	1.7	19	1	94.7	1.8	1.7
30	3	1	66.7	3.5	2.2	3	2	33.3	2.1	0.2
31	10	1	90.0	1.9	1.6	10	5	50.0	1.9	0.7
32	1	0	100.0	4.1	4.1	1	0	100.0	2.3	2.3
33	11	2	81.8	2.6	2.2	10	3	70.0	2.3	1.6
34	11	1	90.9	1.7	1.5	11	2	81.8	2.1	1.5
35	9	0	100.0	1.8	1.8	9	2	77.8	1.7	1.2
36	1	0	100.0	2.8	2.8	1	0	100.0	2.2	2.2
37	1	0	100.0	2.5	2.5	1	1	0.0	2.5	-0.4
38	1	1	0.0	4.1	-0.2	1	1	0.0	4.1	-0.2
39	2	1	50.0	2.0	0.6	2	0	100.0	1.7	1.7
40	2	0	100.0	1.9	1.9	1	0	100.0	2.0	2.0
41	5	1	80.0	1.5	1.0	5	2	60.0	2.2	1.2
42	2	1	50.0	3.4	0.8	2	0	100.0	1.9	1.9
43	1	0	100.0	2.6	2.6	1	0	100.0	1.1	1.1
44	1	1	0.0	3.0	-0.3	1	1	0.0	3.0	-0.3
45	1	1	0.0	3.0	-0.3	1	1	0.0	3.0	-0.3
46	1	0	100.0	2.0	2.0	1	0	100.0	2.0	2.0
47	5	0	100.0	1.9	0.4	4	0	100.0	4.1	4.1
48	2	0	100.0	2.7	2.7	2	2	0.0	3.2	-0.4
49	4	0	100.0	2.0	2.0	3	0	100.0	1.7	1.7
50	3	1	66.7	4.1	2.7	2	1	50.0	4.1	1.9
51	4	1	75.0	2.5	1.9	4	1	75.0	3.0	2.0
52	2	0	100.0	3.2	3.2	2	1	50.0	6.2	3.0
53	24	3	87.5	3.5	2.7	24	6	75.0	2.8	2.0
54	2	1	50.0	3.1	0.6	1	1	0.0	4.8	-0.2
55	20	7	65.0	4.2	2.5	20	2	90.0	3.2	2.8
56	7	1	85.7	2.8	2.4	6	1	83.3	3.0	2.2

© 2025 Chung KC et al. *JAMA Network Open*.

57	11	6	45.5	4.4	1.1	11	7	54.5	2.9	0.8
58	6	2	66.7	2.9	1.8	5	3	40.0	4.4	0.6
59	5	0	100.0	2.7	2.7	4	2	50.0	2.9	0.7
60*	2	0	100.0	1.9	1.9	1	1	0.0	2.3	-0.4
61*	7	3	57.1	2.4	1.1	6	0	100.0	2.1	1.5
62*	8	6	25.0	2.6	0.5	7	3	57.1	3.0	1.4
63*	7	1	85.7	2.7	2.3	6	2	66.7	2.1	1.3
64*	10	4	60.0	3.9	2.1	9	2	77.8	2.8	2.0
65*	14	3	78.7	2.6	1.9	14	3	78.7	2.9	2.0
**mean**	**5.3**	**1.6**	**64.6**	**2.8**	**1.4**	**4.9**	**1.4**	**66.0**	**2.7**	**1.5**

Supplemental Table 2. Frequency of complications overall and in the second half of each surgeon's cases.

Complication	Number of digits (total) (out of 653 digits)	Number of patients (second half) (out of 306 digits)
Any complication	438	96
1		
2	333	162
3	83	42
4	18	4
	4	1
Stiffness	221	104
Nonunion	15	8
Severe infection	16	6
Any revision	193	77

© 2025 Chung KC et al. *JAMA Network Open*.
