# Supplementary material for: Proficiency and Difficulty Scoring Tools for Finger Replantation
Source: JAMA Netw Open. 2025 Oct 30;8(10):e2540453. doi: 10.1001/jamanetworkopen.2025.40453 (PMC12576492; doi:10.1001/jamanetworkopen.2025.40453)
Supplement: Supplement 2. — Data Sharing Statement [file jamanetwopen-e2540453-s002.pdf]

Data Sharing Statement

Chung. Proficiency and Difficulty Scoring Tools for Finger Replantation. *JAMA Netw Open*. Published October 30, 2025. doi:10.1001/jamanetworkopen.2025.40453

Data
Data available: No
